# Treatment of Pseudomonas and *Staphylococcus* Bronchopulmonary Infection in Patients with Cystic Fibrosis

**DOI:** 10.1155/2013/645653

**Published:** 2013-12-30

**Authors:** Rashmi Ranjan Das, Sushil Kumar Kabra, Meenu Singh

**Affiliations:** ^1^Department of Pediatrics, All India Institute of Medical Sciences, Bhubaneswar 751019, India; ^2^Department of Pediatrics, All India Institute of Medical Sciences, New Delhi 110029, India; ^3^Department of Pediatrics, Postgraduate Institute of Medical Education and Research, Chandigarh 160012, India

## Abstract

The optimal antibiotic regimen is unclear in management of pulmonary infections due to pseudomonas and *staphylococcus* in cystic fibrosis (CF). We systematically searched all the published literature that has considered the evidence for antimicrobial therapies in CF till June 2013. The key findings were as follows: inhaled antipseudomonal antibiotic improves lung function, and probably the safest/most effective therapy; antistaphylococcal antibiotic prophylaxis increases the risk of acquiring *P. aeruginosa*; azithromycin significantly improves respiratory function after 6 months of treatment; a 28-day treatment with aztreonam or tobramycin significantly improves respiratory symptoms and pulmonary function; aztreonam lysine might be superior to tobramycin inhaled solution in chronic *P. aeruginosa* infection; oral ciprofloxacin does not produce additional benefit in those with chronic persistent pseudomonas infection but may have a role in early or first infection. As it is difficult to establish a firm recommendation based on the available evidence, the following factors must be considered for the choice of treatment for each patient: antibiotic related (e.g., safety and efficacy and ease of administration/delivery) and patient related (e.g., age, clinical status, prior use of antibiotics, coinfection by other organisms, and associated comorbidities ones).

## 1. Background

Cystic fibrosis (CF) is the most frequent life-threatening congenital disease in Caucasians. Airway colonization with pathogens like *P. aeruginosa* and *S. aureus* belongs to the primary reasons for premature death in patients with CF and antibiotic treatment is a primary reason for improvement of life expectancy within the last decades in patients treated with aggressive antimicrobial regimes [1]. Thereby, CF patients in middle Europe and the US did not reach school age some decades ago, and now CF patients in these countries survive for about 40 years by mean [1]. Therefore the question to optimize antibiotic treatment is a crucial issue in CF care and, basically, the approach of a survey on evidence based antimicrobial therapy in CF can give some—but possibly limited—answers to this basal question. The objective of this systematic review is to summarize the available evidence on the use of antibiotics for the treatment of patients with CF infected by *P. aeruginosa *and *S. aureus* (MSSA, MRSA). We aim to include randomized trials (RCTS) mainly, but if we find no RCT for any of the outcome, we will discuss the observational studies.

## 2. Methods

### 2.1. Search Strategy

We searched the Cochrane Central Register of Controlled Trials (CENTRAL), which contains the Cochrane Cystic Fibrosis Group and the Cochrane Infectious Diseases Group Specialized Registers, MEDLINE (1970 to June 2013). For MEDLINE search, following search terms were adopted: antibiotic route (*oral, intravenous OR nebulised OR inhaled OR aerosol*) *AND* antibacterial agents (*aztreonam OR tobramycin OR colistin or fluoroquinolones OR penicillin OR aminoglycoside OR glycopeptide OR cephalosporin*) AND (*cystic fibrosis*) AND infection (*pseudomonas OR Staphylococcus*) AND (*clinical trial, randomized controlled trial*) AND (*pneumonia OR lower respiratory tract infection*) AND (*child OR children OR infant OR paediatric OR pediatric OR adult*). Two independent reviewers reviewed the search results to identify relevant original human clinical trials. Additional studies were identified through manual searches of reference lists of the originally identified studies as well as reviews on the subject. No language restrictions were applied.

### 2.2. Study Selection

Trials were selected if they used any route for administration of antibiotics for the eradication, prophylaxis, and/or treatment of either* P. aeruginosa *and/or *S. aureus *in patients with CF of any age and both sexes, treatment allocation was randomized or quasirandomized, and there was a control group (placebo or another inhaled antibiotic) and studied clinical (with or without microbiological) parameters. Trials only reporting microbiological parameters were excluded. In case there was no RCT for an important outcome, we considered observational studies if available.

### 2.3. Search Results

A Cochrane Library (CENTRAL) search using the term “*antibiotics*” and filter “*Record Title*” yielded 26 Cochrane Systematic Reviews (CSR) and 1 protocol, 15 other (systematic) reviews, and 124 clinical trials. Simultaneous PUBMED search using the above search terms *yielded* 178 trials. Hand searching of the bibliography of relevant citations yielded an additional 32 papers that were retrieved and examined.

## 3. Results

After applying above exclusion criteria (under study selection), removing the duplicates, and excluding review papers, 208 references were obtained. These were reviewed again in order to determine if they met the selection criteria. One hundred and seventy-four references were discarded: 77 were not randomized/quasirandomized (for an outcome with already existing RCTs), 38 on patients without CF, 21 substudies, 19 pharmacokinetic studies, 12 pharmacodynamic studies, 10 in vitro studies, and 7 pharmacoeconomic studies. Finally, 24 RCTs (*P. aeruginosa* = 21; *S. aureus* = 3), and 10 observational studies (*P. aeruginosa* = 3; *S. aureus* = 7) were included in the present review. Please also refer to Tables 1–6 for characteristics of the studies and summarization of key results. First, the antibiotic strategy about eradication of first or new airway colonization and treatment of chronically persistent airway colonization with *P. aeruginosa* will be discussed followed by the strategy for chronic suppressive therapy and eradication of *S. aureus* (both MSSA and MRSA).

### 3.1. *Pseudomonas aeruginosa*

Individuals with cystic fibrosis (CF) whose respiratory tract is colonized with *P. aeruginosa* have as a group increased pulmonary disease, a more rapid decline in pulmonary function and a decreased survival to adulthood. Studies have shown that antibiotic therapy initiated shortly after a new detection of *P. aeruginosa *is effective in preventing or delaying the onset of chronic infection. Antibiotics administered via parenteral, inhaled, and oral routes are efficacious; however, the optimal regimen and duration of therapy remain unclear. Inhaled antibiotics are an attractive option, delivering high concentrations of antibiotic directly to the infection site while minimizing systemic exposure. In the present review, we will discuss evidence based antimicrobial therapy of *P. aeruginosa *in two parts: first part consisting of eradication of first or new air way colonization and the second part consisting of treatment of chronically persistent airway colonization.

### 3.2. Eradication of First or New Airway Colonization with *P. aeruginosa*

The effectiveness of the various antibioticregimes in eradicating early *P. aeruginosa* requires careful evaluation. There are many *P. aeruginosa* eradication protocols which utilize inhaled/nebulized or intravenous (iv) antipseudomonal antibiotics with or without oral antibiotics. These regimens are discussed below.

### 3.3. Tobramycin (Inhaled) versus Placebo or Other Antibiotics

A Cochrane review (CR) [36] including 2 RCTs [37, 38], and another new RCT [39] were analyzed. Evidence from two trials [37, 38] showed treatment of early *P. aeruginosa* infection with inhaled tobramycin results in microbiological eradication of theorganism from respiratory secretions more often than placebo (OR 0.15 (95% CI 0.03 to 0.65)) and that this effect may persist for up to 12 months. In a recent trial [39], 58 patients with median age of 9 years were randomized to treatment with tobramycin inhalation solution (TIS) for 28 days or inhaled sodium colistimethate (2 × 2 million units/d) plus oral ciprofloxacin (30 mg/kg/day) for 3 months (CC). The authors found no difference, and the two treatment groups resulted in similar eradication success at the end of treatment (80 and 90%, resp.) and similar clinical evolution during the first 2 years of follow-up.

In a cohort study [40], 15 patients (mean age 9 years) inhaled 80 mg tobramycin twice daily (BID) for 12 months. After 1 year, 14/15 was free from *P. aeruginosa*, and after 2 years, 9/15 had negative serum antibody titers against *P. aeruginosa*. There was an improvement in lung function noted before the intervention. In another cohort study [41], 36 young children treated with TSI (tobramycin solution for inhalation) 300 mg BID for 28 days or 56 days eradicated *P. aeruginosa* for up to 3 months after treatment.

There are three new trials [42–44] that were not included in the above CR. The ELITE trial [42] included 88 subjects and used microbiological (not clinical) criteria as the primary outcome. The authors found that treatment with TIS for 28 days is an effective and well-tolerated therapy in CF. The larger EPIC study [43] included 304 children. Participants randomized to cycled therapy received TIS for 28 days, with oral ciprofloxacin or oral placebo for 14 days every quarter, while participants randomized to culture-based therapy received the same treatments only during quarters with positive *P. aeruginosa *cultures. There were no statistically significant differences in exacerbation rates between cycled and culture-based groups (hazard ratio (HR), 0.95; 95% CI, 0.54–1.66) or ciprofloxacin and placebo (HR, 1.45; 95% CI, 0.82–2.54). The odds ratio (OR) of *P. aeruginosa* positive culture comparing the cycled versus culture-based group was 0.78 (95% CI, 0.49–1.23) and 1.10 (95% CI, 0.71–1.71) comparing ciprofloxacin versus placebo. The Italian EPIC study [44] included 263 subjects to clarify the efficacy of two different eradication treatments, oral ciprofloxacin, and TIS (test treatment), compared with oral ciprofloxacin and inhaled colistin (reference treatment). Hundred five patients were assigned to inhaled colistin/oral ciprofloxacin (arm A) and 118 were assigned to inhaled tobramycin/oral ciprofloxacin (arm B). *P. aeruginosa* was eradicated in 66 (62.8%) patients in arm A and in 77 (65.2%) in arm B (OR 0.90, 95% CI 0.52 to 1.55). Following treatment, an increase in *S. maltophilia* was noted (OR 3.97, 95% CI 2.27 to 6.94) with no differences between the two arms (OR 0.89, 95% CI 0.44 to 1.78).

### 3.4. Colistin versus Placebo

We could identify one study [45]. This cohort study including very few patents (*N* = 7) with recent *P. aeruginosa *positive cultures used inhaled Colomycin 500,000 U BID and found a 36% reduction in the culture rate in long term.

### 3.5. Ciprofloxacin and Colistin versus Control

One RCT [46] and three cohort studies [47–49] were included. The RCT by Valerius et al. [46] included 26 participants and used oral ciprofloxacin (250–750 mg BID) and inhalations of colistin (1 million units BID) for 3 weeks. During the 27 months of the trial, infection with *P. aeruginosa* became chronic in significantly fewer treated subjects than untreated subjects (14% versus 58%). Frederiksen et al. [47] included 91 participants and used oral ciprofloxacin (25–50 mg/kg/d) and inhalations of colistin (1 million units BID) for 3 weeks. The study was carried out over 44 months as only 16% of the treated patients developed chronic *P. aeruginosa* infection after 3(1/2) years compared with 72% of the control patients. Hansen et al. [48] included 146 patients and used oral ciprofloxacin (25–50 mg/kg/d) and inhalations of colistin (2 million units TID) for 3 months. A Kaplan Meyer plot showed protection from chronic infection in up to 80% of patients for up to 15 years. Treatment failure (*P. aeruginosa* positive culture immediately after the end of treatment of first ever isolate) was a strong risk factor for development of chronic infection after 3-4 years (odds ratio (OR) 5.8). Schelstraete et al. [49] included 41 patients and used oral ciprofloxacin (30 mg/kg/d) and inhalations of colistin (2 million units BID) for 3 months. Eleven patients became chronically colonized during the study period over 5 years.

### 3.6. Ciprofloxacin, Colistin, and Tobramycin versus Control

Vazquez et al. [50] included 16 patients and used oral ciprofloxacin (30–40 mg/kg/d) for 2 weeks, inhalations of colistin (1 million units), and inhaled tobramycin (100 mg BID) for long term. In follow-up, *P. aeruginosa* culture was positive in 4.6% of the treatment group compared to 86% of historic control group.

### 3.7. Intravenous (IV) Antibiotics with or without Inhaled and/or Oral Antibiotics

In a pilot study [51] in 28 patients aged from 2 to 18 years, the authors gave a two-week course of azlocillin (150 mg/kg/d) and tobramycin (10–15 mg/kg/d). The eradication of *P. aeruginosa* that was achieved in 18 children was only temporary. Samples from only 10 and 5 patients remained negative 3 and 6 months after treatment, respectively. Only 5 children remained free from *P. aeruginosa* for a prolonged period from 14 to 32 months. Munck et al. [52] initiated treatment with a combination of IV ceftazidime (300 mg/kg/d) or imipenem (75 mg/kg/d) plus tobramycin (7.5 mg/kg/d) for 18–21 days, followed by nebulized colistin (1–3 million units) for >2 months in 19 patients. Initial colonization was eradicated in all patients, but again all reacquired *P. aeruginosa* within 3–25 months during 3 years of follow-up. Griese et al. [53] included 17 patients and used inhaled tobramycin (80 mg BID) for 4 weeks in <5 yrs and ciprofloxacin plus inhaled colistin (1 million units BID) for 3 weeks in >5 yrs. In some patients, IV ceftazidime and tobramycin were also used. Initial *P. aeruginosa* colonization was successfully eradicated in 15 of 17 patients for at least two years. Nixon et al. [54] included 24 patients and used IV ticarcillin clavulanate plus tobramycin for 2 weeks, followed by oral ciprofloxacin or inhaled tobramycin for 3 months. Initial *P. aeruginosa* colonization was successfully eradicated in 25% patients only. Douglas et al. [55] included 26 patients and used IV ticarcillin clavulanate (300 mg/kg/d) or ceftazidime (150 mg/kg/d) plus IV tobramycin (7.5 mg/kg/d) for 2 weeks, followed by oral ciprofloxacin (10 mg/kg BID) and inhaled tobramycin (80 mg/kg BID) for 4 weeks. Initial *P. aeruginosa* colonization was successfully eradicated in 23 of 26 patients, and 3 of 23 patients developed recurrences after 1 year.

### 3.8. Treatment of Chronically Persistent Airway Colonization with *P. aeruginosa*

It has been seen that the long-term effect on the prevalence ofchronic *P. aeruginosa* infection depends on the rate of acquisition of new infections,the efficiency of the eradication regime, that is, theclearance rate, as well as the time period free of *P. aeruginosa* after-treatment. Recent data shows that the effects of chronicinfection are more severe in those who acquired it at an earlier age [56]. There are many protocols which utilize inhaled/nebulized or oral or intravenous (i.v.) antipseudomonal antibiotics for treatment of chronic infection. These regimens are discussed below.

## 4. The Role of Inhaled Antibiotics 

A Cochrane review studied the role of inhaled antibiotics for long-term suppression of chronic *P. aeruginosa* infection [57]. Seventeen trials including 1562 participants compared an inhaled antibiotic with placebo or usual treatment for a period of 1 and 32 months. Lung function (FEV1) was higher and exacerbations were less in the antibiotic-treated group. Resistance to antibiotics and minor side effects were more in the antibiotic-treated group.

### 4.1. Aztreonam Lysine (AZLI, Inhaled) versus Placebo

For more details, see Table 1.

### 4.2. Aztreonam Lysine (AZLI, Inhaled) versus Tobramycin (Inhaled)

An open label, parallel group trial compared AZLI and tobramycin nebulizer solution (TNS) in 273 patients (≥6 years) [58]. Patients were randomized to three 28-day courses (AZLI 75 mg TID or TNS 300 mg BD); 28 off-days separated each course. Mean baseline FEV1 was 52% predicted. Mean relative changes after 1 course (AZLI: 8.35%; TNS: 0.55%; *P* < 0.001) and mean actual changes across 3 courses (AZLI: 2.05%; TNS: −0.66%; *P* = 0.002) indicated AZLI to be statistical superior over TNS. AZLI-treated patients had fewer respiratory hospitalizations (*P* = 0.044) and respiratory events requiring additional antibiotics (*P* = 0.004).

### 4.3. Tobramycin Inhaled versus Placebo

For more details, see Table 2.

### 4.4. Fosfomycin/Tobramycin versus Placebo

A single RCT evaluated fosfomycin/tobramycin for inhalation (FTI), 160/40 mg or 80/20 mg BID in 119 patients aged ≥18 years versus placebo, for 28 days [59]. The inclusion criteria were chronic *P. aeruginosa* infection and FEV1 25–75%. The authors found reduced rate of respiratory events (dyspnea and wheezing) more with FTI than placebo and more with an 80/20 mg dose of FTI than 160/40 mg dose. No clinically significant differences between groups were reported for laboratory values. FTI maintained the substantial improvements in FEV1% predicted and was well tolerated.

### 4.5. Tobramycin versus Colistin

In a multicenter trial, 115 patients aged ≥6 years were randomised to receive either nebulized tobramycin (TNS) or colistin, BID for 4 weeks [60]. The primary end point was a change in FEV1% predicted. TNS produced a mean 6.7% improvement in lung function (*P* = 0.006), whilst there was no significant improvement in the colistin-treated patients (mean change 0.37%). In another randomized trial, 380 patients aged ≥6 years were randomised to Colobreathe dry powder for inhalation (CDPI, one capsule containing colistimethate sodium 1 662 500 IU, BID) or three 28-day cycles with BID 300 mg tobramycin (TIS) for 24 weeks [61]. The conclusion was that CDPI demonstrated efficacy by virtue of noninferiority to TIS in lung function after 24 weeks.

### 4.6. Colistin Inhaled versus Placebo

For more details, see Table 3.

### 4.7. Other Inhaled Antibiotics versus Placebo

One RCT assessed the efficacy and safety of a novel aerosol formulation of levofloxacin (MP-376, Aeroquin) in 151 patients with CF with chronic *P. aeruginosa* infection [62]. The participants received one of three doses of MP-376 (120 mg OD, 240 mg OD, 240 mg BID) or placebo for 28 days. The authors found a dose-dependent increase in FEV1, with a difference of 8.7% between the 240 mg BID group and placebo (*P* = 0.003). Also a significant reduction (61–79%) in the need for other antimicrobials was observed with all MP-376 treatment groups. In a crossover study, the authors included 20 participants of 15–42 years age and administered carbenicillin (1 g) and gentamicin (80 mg) BID for 6 months [63]. Compared to placebo, improvement in lung function (FEV1, FVC, and PEF) was more and exacerbations of infection (courses of IV antibiotics) were less in treatment group. In another crossover study, the authors included 33 participants of  7.8–16 years age, and administered gentamicin (20 mg) BID for 12 months [64]. There was no significant difference in antibiotic usage, days in hospital or clinical symptoms between no treatment and treatment group, but subjects in treatment group with *P. aeruginosa* in sputum showed significantly less deterioration in lung function over 2 years. Yet, in another crossover design, the authors included 7 participants with mean age of 15.6 years and administered gentamicin (80 mg) TID for 3 months [65]. There was no significant difference in the lung function (FEV1, FVC) between the two groups.

A randomized crossover study compared three treatment groups: ceftazidime, gentamicin and carbenicillin, and saline, each given for 4 months [66]. There was significant improvement in the lung function (PEF, FEV1, and FVC) in both the treatment groups compared to the saline group, but there was no difference in the two treatment group.

## 5. The Role of Systemic Antibiotics 

### 5.1. Oral Fluoroquinolones Compared to Placebo or Other Antibiotics

In a RCT, 31 participants of  ≥18 years of age received ciprofloxacin or placebo for 10 days every 3 months for 1 year [67]. In the treatment group, patients reported a significant improvement in cough and PEF but not in the FEV1 and FVC. Also, there was no reduction in the hospital admissions or the number of courses of IV antibiotics.

In a randomized trial including participants of 8–25 years of age, 21 were randomly assigned to oral ciprofloxacin alone and 23 were randomly assigned to ciprofloxacin plus inhaled amikacin [68]. Continued improvement in clinical symptoms was observed in 14 patients in both treatment groups and the difference was not significant.

In a randomized crossover study 26 adult patients received ciprofloxacin 750 mg BID or ofloxacin 400 mg BID for 14 days, with three months washout period [69]. Treatment with both the drugs was associated with improvement in the clinical score, lung function tests, and inflammatory parameters; no difference between ciprofloxacin and ofloxacin was found.

In an open prospective clinical trial, the clinical efficacy of the conventional aminoglycoside plus beta-lactam treatment was compared to that of monotherapy with oral quinolones in 26 adult patients [70]. Six two-week courses of antipseudomonas treatment were administered with an interval of approximately three months between treatments. In each patient, two courses of conventional treatment were followed by two courses of quinolone treatment and then by other two courses of conventional treatment. The observed improvements in pulmonary function were somewhat higher when the patients received conventional treatments, and in the most seriously affected patients, conventional treatment was significantly better than quinolone treatment.

### 5.2. Azithromycin Compared to Placebo or Other Antibiotics

For more details, see Table 4.

### 5.3. Parenteral Antibiotics Compared

Six children with *P. aeruginosa* isolated from their respiratory tract completed a randomized crossover study of oral flucloxacillin and nebulized aminoglycoside versus placebo [71]. The patients in the treatment group had higher FEV1 results at the end of the month of active treatment than placebo.

In a prospective multicenter interventional trial of iv meropenem (120 mg/kg/day) or iv ceftazidime (200–400 mg/kg/day), each administered together with iv tobramycin (9–12 mg/kg/day) and 78 patients were included for suppression therapy of chronic *P. aeruginosa* colonization [72]. Both treatments improved lung function, and no difference between treatment groups was observed.

### 5.4. *Staphylococcus aureus*

*S. aureus* is one of the first microbes and also one of the commonest to infect patients with cystic fibrosis. There has been an increase in the prevalence of colonization/infection with both methicillin-susceptible (MSSA) and methicillin resistant (MRSA) *S. aureus* over the past decade [73]. Colonization of the anterior nares with *S. aureus* represents an important risk factor for subsequent infection in both healthy and diseased population, but only few studies have investigated colonization in with CF. In one study, the authors reported a significantly increased prevalence among patients with CF who had not received anti-staphylococcal prophylaxis prior to taking the cultures [74]. Another study using nasal lavage found the presence of identical genotypes in upper and lower airways, which suggests that upper airways play a role as a reservoir of *S. aureus* (like *P. aeruginosa*) in CF [75]. In a 2-year cohort study of 100 children with CF, small-colony variants (SCVs) of *S. aureus* were detected among 24% of participants and were significantly associated with a greater drop in lung function during the study [76]. Other studies have also found SCVs to be associated with higher rates of antimicrobial resistance and more advanced lung disease [77]. We will discuss below the treatment (prophylactic and eradication) strategy for *S. aureus.*

### 5.5. Methicillin Sensitive *S. aureus* (MSSA)

The approach for eradication of an initial infection and chronic suppressive treatment are different. In a retrospective cohort study, the authors enrolling 191 patients reported eradication of MSSA in 74% of the subjects after a single course of anti-staphylococcal antibiotics [78]. With continuing treatment, only 9% were found to be chronically infected over a six-month period, and on further follow-up, only a low level of resistance was found to anti-staphylococcal antibiotics [79]. Based on this, the European CF Consensus group has recommended initial 2–4 weeks of anti-staphylococcal antibiotic with new *S. aureus* infection [80]. However, the long-term results of such a approach are unknown and warrant further investigation.

Regarding the early chronic suppressive therapy, there have been many studies with variable results. These are summarized in Table 5.

As it can be seen from the table, early chronic suppressive treatment of *S. aureus* has been associated with an increased infection with *P. aeruginosa* without any major clinical benefits. Same was the findings by the Cochrane review [81]. Though the US Guidelines do not recommend use of prophylactic anti-staphylococcal antibiotics as the UK and Australian guidelines, however, recommend flucloxacillin prophylaxis starting from the infancy [82].

Like treatment of *P. aeruginosa *with inhaled antibiotics, few studies have the role of inhaled antibiotics in the chronic treatment of MSSA infection. In one study, 13 patients (3–34 years) with chronic bronchopulmonary infection due to MSSA were treated with nebulized ampicillin (500 mg/12 h in those weighing <40 kg and 1 g/12 h in those >40 kg) over a period from 6 to 45 months (mean, 23 months) [83]. A significant reduction in the consumption of oral antibiotics (from 28 to 7 days/year) and number of hospitalizations (from 4 to 1/year) were observed. No significant differences were found for lung function, although it did not decline during the entire treatment period. Neither there was co-colonization due to *P. aeruginosa* nor was MSSA eradicated.

### 5.6. Methicillin Resistant *S. aureus* (MRSA)

There are no current guidelines for treatment of MRSA in patients with CF. The prophylactic treatment has its own problem of emergence of antimicrobial resistance without any appreciable long-term effect. The treatment regimen differs depending upon whether outpatient or inpatient therapy is indicated. Drugs used for outpatient therapy include co-trimoxazole, minocycline (in children > 8 years) and linezolid. If inpatient therapy is indicated, then iv vancomycin or teicoplanin are the drugs of choices. Recently, inhaled drugs like tobramycin/fosfomycin and inhaled vancomycin have been tried with some success [84].

Regarding the eradication protocol, there have been few uncontrolled studies done so far. These are summarized in Table 6.

Though the concerns about MRSA and the success with early *P. aeruginosa* eradication have encouraged several centers to attempt eradication of MRSA, the long-term results are unknown. We need long-term controlled follow-up studies before any recommendations/guidelines can be made regarding the same.

## 6. Discussion

### 6.1. Key Findings

*P. aeruginosa* colonisation has a negative effect on lung function in patients with cystic fibrosis (CF). It is rather easy to eradicate the organism in the early stage of colonisation and to maintain a reduced bacterial density during chronic colonisation. For this, intermittent (few monthly) microbiological culture is advisable. Once the organism is isolated, the therapy depends upon presence or absence of symptoms. As a guide, the first isolation of *P. aeruginosa* without any clinical signs should be treated with oral ciprofloxacin plus inhaled aztreonam (AZLI) or colistin (COL) or tobramycin (TOB) (alternative being iv treatment with or without inhaled antibiotics) [85]. Reviewing the available data on the efficacy and safety of aztreonam (AZLI), colistin (COL), and tobramycin (TOB) administered by inhalation, we have discussed significant differences among these antibiotics. Inhaled antipseudomonal antibiotic treatment improves lung function. However, more evidence, from trials of longer duration, is needed to determine whether this benefit is maintained and to determine the significance of development of antibiotic-resistant organisms. Regarding the maintenance treatment of chronic *P. aeruginosa* infection/colonization, stable patients > 6 years of age should be treated with any one of the inhaled antibiotics. For patients with development of mild symptoms, oral ciprofloxacin, and those with severe symptoms, intravenous antibiotics (preferably in combination) can be added [85]. Patients with highly resistant pathogens detected in sputum cultures may still derive clinical benefits from aerosolized antibiotics. This may be due to the substantial pharmacodynamic benefits of aerosolized antibiotics; that is, high concentrations of drug can be delivered to the site of infection with low risk of toxicity.

*S. aureus* is one of the earliest bacteria to be detected in infants and children with CF. The rise of MRSA in the last decade has caused a lot of attention to this organism, as the isolation of this organism has been associated with a decline in lung function. Similar to *P. aeruginosa*, many centers target this organism for aggressive treatment because of the negative impact on CF patients. As we have already discussed, there are many therapeutic options for both MSSA and MRSA. But many questions remain regarding the clinical utility and tradeoffs of prophylactic therapy for MSSA and eradication and treatment for MRSA. We also highlighted paucity of RCTs in the therapy of *S. aureus*. In order to advance the care of CF patients, controlled clinical trials are needed to find the optimal approach for managing CF patients who are infected with either MSSA or MRSA. But, currently no consensus exists regarding the same.

### 6.2. Limitations

A number of limitations apply to all the trials (mostly RCTs) included in this overview. First, most of the trials included relatively small numbers of patients, which lack of adequate power to prove the hypothesis (outcome measures). There is a probability of a type II error, simply because of the comparable study sizes and the limited number of studies, therefore population size under review. Second, not all trials are reported on each key outcome and outcomes are not reported in a consistent format.

### 6.3. Direction for Future Research

As the inflammatory response of airways and the effect of inhaled antibiotics may not be the same in children and adults and many CF patients are surviving beyond adolescence, age-stratified analyses should be performed in future clinical trials. Increased availability of new inhaled antibiotics should also allow comparative trials to be performed between them. Though, assessment of pulmonary function (FEV1) is the common end point in many trials, quality of life (symptom score, medication score, and level of bother) should also bemeasured. Besides the standardtreatment regimen with 28-day on/28-day off cycles of inhaled antibiotics, feasibility of easier delivery schedules (such as 1 or 2 week on/off cycles or once daily dosing) should be investigated [86].

## 7. Conclusions

As it is difficult to establish a firm recommendation based on the available evidence, the following factors must be considered for the choice of treatment for each patient: antibiotic related (e.g., safety and efficacy and ease of administration/delivery) and patient related factors (e.g., age, clinical status, prior use of antibiotics, coinfection by other organisms, and associated comorbidities).

## Figures and Tables

**Table 1:** Summary of AZLI trials.

Author	Study design	Subjects	Dose used	Outcomes	Main results
McCoy et al. [2]	Multicenter, RCT.	*N* = 246. Age = >6 years. Inclusion criteria: *P. aeruginosa*, 3 or more courses of tobramycin in previous year, FEV1 ≥25% and ≤75% predicted	Aztreonam 75 mg for 4 weeks, BID or TID	Time to need for additional antibiotics, FEV1	Increased time to need for additional antibiotics, improved FEV1

Retsch-Bogart et al. [3]	Multicenter, RCT	*N* = 105. Age = >13 years. Inclusion criteria: mild and moderate lung disease, and no recent use of antibiotics, FEV1 ≥40%	Aztreonam 75 or 225 mg BID	Percent change in FEV1 at end of 14 days	No significant change in FEV1, trend of greater improvement in lung function in those with worse baseline FEV1

Retsch-Bogart et al. [4]	Multicenter, RCT	*N* = 164. Age = >6 years. Inclusion criteria: moderate-to-severe lung disease, FEV1 ≥25% and ≤75% predicted	Aztreonam 75 mg TID for 28 days	Change in patient-reported respiratory symptom score	Significant improvement in self-reported symptom scores, improved FEV1

Oermann et al. [5]	Open label follow-up study over 18 months	*N* = 274. Age = >8 years. Inclusion criteria: previous participant in two other studies [2, 5]	Dose used in the main trials [2, 5]	Safety and efficacy	Improved FEV1 and symptom scores, at the end of each cycle

Wainwright et al. [6]	Multicenter, RCT	*N* = 157. Age = ≥6 years. Inclusion criteria: FEV1 >75%	Aztreonam 75 mg TID for 28 days	Change in patient-reported respiratory symptom score	No significant change in symptom score, improved FEV1

**Table 2:** Summary of TIS/TSI trials.

Author	Study design	Subjects	Dose used	Outcomes	Main results
Chuchalin et al. [7]	Multicenter, RCT	*N* = 247. Age = Adults. Inclusion criteria: chronic *P. aeruginosa* infection	Tobramycin 300 mg for 24 weeks	Percent change in FEV1, FVC, and FEF25–75%, pulmonary exacerbations, use of parenteral antibiotics, and rate of hospitalizations	Significantly improved FEV1, FVC, and FEF25–75%. The % of patients hospitalized as well as the need for parenteral antibiotics was significantly lower

Lenoir et al. [8]	RCT	*N* = 59. Age = 6–30 years. Inclusion criteria: chronic *P. aeruginosa* infection	Tobramycin 300 mg BID for 4 weeks	Percent change in FEV1, FVC, and FEF25–75%	Significantly improved FEV1, FVC, and FEF25–75%

MacLusky et al. [9]	RCT	*N* = 28. Age = 7–24 years. Inclusion criteria: chronic *P. aeruginosa* infection	Tobramycin 80 mg BID for 33 months	Lung function (FEV1 and FVC), clinical scores, and exacerbations	The treatment group showed no change, while the control group had a significant decline in both pulmonary function and clinical status

Murphy et al. [10]	Multicenter, RCT	*N* = 184. Age = 6–15 years. Inclusion criteria: CF with mild lung disease	Tobramycin 300 mg BID, alternating 4-weekly cycles for 56 weeks	Lung function, hospitalisation, and antibiotic use	Significant reductions in hospitalizations, antibiotic use, and a trend towards improvement in FEF25–75%

Ramsey et al. [11]	Multicenter, Crossover study	*N* = 71. Age = ≥6 years. Inclusion criteria: chronic *P. aeruginosa* infection and FVC >40%	Tobramycin 600 mg TID for 4 weeks then crossover for two 28-day periods	Lung function (FEV1, FVC, and FEF25–75%), exacerbations of infection and antibiotic use	Increase in the % change in FEV1, FVC, and FEF25–75%. Fewer exacerbations of infection and antibiotic use

Ramsey et al. [12]	Multicenter, RCT	*N* = 520. Age = ≥6 years. Inclusion criteria: chronic *P. aeruginosa* infection, FEV1 ≥25% and ≤75% predicted	Tobramycin 300 mg BID in three on-off cycles for a total of 24 weeks	Lung function (FEV1 and FVC), exacerbations (hospitalization or IV antibiotics)	Increase in the % change in FEV1 and FVC. Fewer hospitalizations and antibiotic use

Moss [13]	Multicenter, RCT	*N* = 128. Age = 13–17 years. Inclusion criteria: chronic *P. aeruginosa* with mild-to-moderate lung disease (FEV1 ≥25% and ≤75% predicted)	Tobramycin 300 mg in three 28-day cycles	Pulmonary function, incidence of hospitalization, and IV antibiotic use	Increase in the % change in FEV1. The average number of hospitalizations and IV antibiotic courses did not increase over time

Stelmach et al. [14]	Observational study	*N* = 12. Age = 6–18 years. Inclusion criteria: chronic *P. aeruginosa *infection with FEV1 ≥25% and ≤75% predicted	Tobramycin 300 mg in two 28-day cycles	Pulmonary function, clinical status over 2-year period	Significant decline in lung function, clinical improvement

Galeva et al. [15]	RCT	*N* = 62. Age = 6–21 years. Inclusion criteria: chronic *P. aeruginosa *infection and FEV1 ≥25% and ≤80% predicted	Tobramycin BID for one treatment cycle (18.5 days on drug, 28 days off drug)	Change in FEV1%, quality of life	Change in FEV1%, quality of life

Konstan et al. [16]	RCT	*N* = 95. Age = 6–21 years. Inclusion criteria: chronic *P. aeruginosa *infection and FEV1 ≥25% and ≤80% predicted	Tobramycin 112 mg BID for a total of three cycles (each cycle, 28 days on and 28 days off drug)	Change in FEV1%	Increase in FEV1% along with decrease in the number of hospitalizations and antibiotic use

**Table 3:** Summary of inhaled colistin trials.

Author	Study design	Subjects	Dose used	Outcomes	Main results
Jensen et al. [17]	RCT	*N* = 40. Age = 7–35 years. Inclusion criteria: chronic *P. aeruginosa* infection	Colistin (1 million units) BID for 3 months	Lung function (FEV1 and FVC) and clinical score	Significant improvement in clinical symptom score and pulmonary function

Day et al. [18]	Crossover study	*N* = 14. Age = 5–16 years. Inclusion criteria: chronic *P. aeruginosa* infection	Colistin (1 million units) BID for 3 months	Lung function (FEV1 and FVC), antibiotic use and hospital admissions, and symptom score	Significantly improved FEV1, FVC. Decreased antibiotic use and hospitalization, and decreased symptom score

Nikonova et al. [19]	Prospective study	*N* = 2. Age = ≤5 years. Inclusion criteria: chronic *P. aeruginosa* infection	Colistin (1 million units) BID for 3 weeks, followed till 1 year	Exacerbation rate, pulmonary symptoms	Significant improvement in the symptoms and reduced exacerbation

**Table 4:** Summary of azithromycin trials.

Author	Study design	Subjects	Dose used	Outcomes	Main results
Wolter et al. [20]	RCT	*N* = 60. Age = 27.9 (±6.5) years. Inclusion criteria: chronic *P. aeruginosa* infection	Azithromycin 250 mg OD for 3 months	% change in FEV1 (FVC), weight, quality of life (QOL), respiratory exacerbations	Improved QOL, reduced number of exacerbations, and reduced rate of decline in lung function

Equi et al. [21]	Crossover study	*N* = 41. Age = 8–18 years. Inclusion criteria: chronic *P. aeruginosa* infection and with a median FEV1 of 61%	Azithromycin 250 mg (500 mg if weight >40 kg) OD for 6 months	% change in FEV1 (FVC and MEF), exercise tolerance, and subjective wellbeing	FEV1 improved by ≥13% and fewer courses of antibiotics were required in the treatment group. Rest of other parameters did not improve.

Saiman et al. [22]	Multicenter, RCT	*N* = 185. Age = ≥6 years. Inclusion criteria: FEV1 of ≥30%	Azithromycin 250 mg (500 mg if weight >40 kg) 3 days/week for 168 days	% change in FEV1, weight, respiratory exacerbations	Significant improvement in the FEV1%, weight, and reduced exacerbations

Clement et al. [23]	Multicenter, RCT	*N* = 82. Age = 6–21 years. Inclusion criteria: FEV1 of ≥40%	Azithromycin 250 mg (500 mg if weight > 40 kg) 3 days/week for 12 months	% change in FEV1 (FVC), weight, respiratory exacerbations, additional antibiotic treatment	FEV1% change did not differ significantly, but the numbers of pulmonary exacerbations and the additional courses of antibiotics were significantly reduced in the treatment group

Steinkamp et al. [24]	Multicenter, RCT	*N* = 38. Age = 24.8 (±10) years. Inclusion criteria: chronic *P. aeruginosa* infection	Azithromycin (<30 kg: 500 mg, 30–39 kg: 750 mg, 40–49 kg: 1000 mg, and ≥50 kg: 1250 mg) once/week for 8 weeks	% change in FEV1 (FVC), weight, quality of life (QOL), respiratory exacerbations	Pulmonary function declined in both the groups, but QOL was improved in the treatment group.

Saiman et al. [25]	Multicenter, RCT	*N* = 260. Age = 6–18 years. Inclusion criteria: FEV1 of at least 50% predicted and negative respiratory tract cultures for *P. aeruginosa* for at least 1 year	Azithromycin 250 mg (500 mg if weight > 36 kg) 3 days/week for 6 months	% change in FEV1, weight, respiratory Exacerbations, treatment requirements	No significant change in the FEV1%, rate of hospitalizations, and use of additional antibiotics. But reduced exacerbations and increased weight in treatment group

**Table 5:** Summary of studies on early chronic suppressive therapy of MSSA.

Author	Study design	Subjects	Drug used	Outcomes	Main results
Loening-Baucke et al. [26]	RCT	*N* = 17. Age = infants and children. Inclusion criteria: CF diagnosis	Cephalexin	Clinical and microbiological	Significant improvement in clinical and microbiological parameters

Weaver et al. [27]	RCT	*N* = 38. Age = 7 weeks (prophylaxis group), 5 weeks for (as required group). Inclusion criteria: CF diagnosis	Oral flucloxacillin 250 mg/day up to 2 years	Clinical and microbiological parameters, hospital admissions	More cough, greater numbers of *S aureus* isolates and increased hospitalization in the other (as required) group

Nolan et al. [28]	Prospective study	*N* = 47. Age = children. Inclusion criteria: mild to moderate CF	Inhaled cephaloridine and oral cloxacillin in one group. Only oral cloxacillin in other groups	Number of respiratory tract infections or hospital admissions and change of pulmonary function	*Haemophilus influenzae* carriage was greater in the group not receiving inhaled antibiotic. High rates of carriage of *P. aeruginosa* and *P. cepacia* in both the groups

Ratjen et al. [29]	Prospective study	*N* = 639. Age = <18 year. Inclusion criteria: *P. aeruginosa* negative prior to entry and at least 2 additional *P*.* aeruginosa* negative respiratory cultures while being followed up	48.2% received continuous prophylaxis, 40.4% received intermittent, and 11.4% received no prophylaxis with anti-staphylococcal antibiotics	Number of respiratory tract infections	Continuous prophylaxis group has a high rate of acquisition of *P. aeruginosa* than the other two groups

Stutman et al. [30]	Multicentre, RCT	*N* = 119. Age = <2 year Inclusion criteria: CF diagnosis	Oral cephalexin 80–100 mg/kg/day up to 5–7 years	Clinical, microbiologic, laboratory, radiographic, and anthropometric outcomes	Except an increased isolation of *P. aeruginosa*, there were no other benefits

**Table 6:** Summary of trials for eradication of MRSA.

Author	Study design	Subjects	Drug used	Outcomes	Main results
Solís et al. [31]	Retrospective	*N* = 12. Age (median) = 117 months. Inclusion criteria: CF with chronic MRSA infection	Continuous oral cephradine along with topical application of oral and nebulized vancomycin for 5 days	Microbiological and pulmonary function	MRSA was eradiated in 55%. FEV1 was not affected

Macfarlane et al. [32]	Prospective	*N* = 17. Age = <18 years Inclusion criteria: CF with chronic MRSA infection	One five-day course of oral rifampicin and fusidic acid ± intravenous teicoplanin	Microbiological	MRSA was eradiated in 47% cases after the first course, in 71% after the second course, and in 94% when teicoplanin was added

Garske et al. [33]	Prospective study	*N* = 7. Age (mean) = 29.3 years. Inclusion criteria: adult CF patients with chronic MRSA infection	Rifampin and oral Fusidate for 6 months	Microbiological, use of iv antibiotics, and change of pulmonary function	MRSA was eradiated in 75%. Reduction in iv antibiotic use without any change in lung function

Halton et al. [34]	Prospective study	*N* = 17. Age = <18 year. Inclusion criteria: CF with chronic MRSA infection	TMP-SMX (Trimethoprim-Sulfamethoxazole) for 4 weeks, mupirocin and rifampin in the last week over 18-months	Microbiological	MRSA was eradiated in 60%

Vanderhelst et al. [35]	Prospective study	*N* = 11. Age (median) = 9 years. Inclusion criteria: CF with chronic MRSA infection	Rifampin and oral Fusidate along with topical mupirocin for 6 months	Microbiological	MRSA was eradiated in 100%
